# Early Dynamics of Portal Pressure Gradient After TIPS Insertion Predict Mortality

**DOI:** 10.1111/apt.18503

**Published:** 2025-01-16

**Authors:** P. A. Reuken, A. Franz, T. H. Wirtz, C. Ripoll, R. Aschenbach, U. Teichgräber, M. R. Pollmanns, M. Kiehntopf, S. Keil, C. Kuhl, P. C. Schulze, C. Trautwein, T. Bruns, A. Stallmach, A. Zipprich

**Affiliations:** ^1^ Department of Internal Medicine IV (Gastroenterology, Hepatology and Infectious Diseases), Jena University Hospital Friedrich‐Schiller‐University Jena Germany; ^2^ Medical Department III University Hospital RWTH Aachen Aachen Germany; ^3^ Department of Radiology Jena University Hospital, Friedrich‐Schiller‐University Jena Germany; ^4^ Institute of Clinical Chemistry and Laboratory Diagnostics Jena University Hospital, Friedrich‐Schiller University Jena Germany; ^5^ Department of Diagnostic and Interventional Radiology University Hospital RWTH Aachen Aachen Germany; ^6^ Division of Cardiology, Angiology and Intensive Medical Care, Department of Internal Medicine I, Jena University Hospital Friedrich‐Schiller‐University Jena Germany

**Keywords:** ascites, cirrhosis, portal pressure gradient, TIPS

## Abstract

**Background:**

Transjugular intrahepatic portosystemic shunt (TIPS) placement leads to a reduction in portal pressure and an improvement in survival in patients with recurrent and refractory ascites and variceal haemorrhage. Prediction of post‐TIPS survival is primarily determined by factors identified before the TIPS procedure, as data collected during or after TIPS implantation are limited. The aim of the study was to evaluate the influence of early hemodynamic changes after TIPS placement on survival, in order to refine post TIPS management.

**Methods:**

In this prospective bicentric study, consecutive patients (*n* = 105) undergoing TIPS placement for ascites or variceal haemorrhage underwent measurement of portal pressure gradient (PPG) immediately at TIPS insertion (PPG0) and 24 h later (PPG24h) and the ΔPPG was calculated from PPG24h and PPG0 (ΔPPG = PPG24h‐PPG0). Kaplan–Meier survival analysis and uni‐ and multivariable regression analyses were conducted to identify survival predictors.

**Results:**

Patients with lack of increased ΔPPG exhibited poorer 90‐day and 1‐year survival compared to patients with increased ΔPPG. This worse survival was independent of The Model for End‐Stage Liver Disease (MELD) score, Child‐Pugh score, bilirubin levels, creatinine and the Freiburg index of post‐TIPS survival (FIPS) > 0.92. Among these patients with poorer outcome, elevated bilirubin (> 25 μmol/L) further distinguished survivors from non‐survivors.

**Conclusion:**

Lack of increased ΔPPG post‐TIPS insertion identifies a high‐risk patient group with worse survival. We propose incorporating this second PPG measurement and determining ΔPPG into clinical practice to identify these patients early and tailor post‐TIPS patient care.

## Graphical abstract

The lack of increase in portal pressure gradients (PPG) within 24 h after TIPS implantation is associated with poorer outcomes.

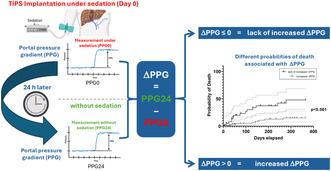

## 1 Introduction

Ascites and variceal haemorrhage represent the predominant decompensating events in patients with cirrhosis [1] and are mainly driven by portal hypertension, which is mainly the consequence of the structural changes of cirrhosis [2, 3]. Transjugular intrahepatic portosystemic shunt (TIPS) constitutes a radiological intervention involving the placement of a stent graft between the portal and hepatic veins leading to an immediate reduction in portal pressure. The placement of TIPS has been associated with survival improvement among patients with refractory ascites [4], recurrent ascites [5] and variceal haemorrhage [6, 7].

A target portal pressure gradient (PPG) of < 12 mmHg following TIPS implantation is sought after, as achieving this pressure threshold is associated with improved survival outcomes and reduced incidences of ascites and variceal bleeding [3, 8]. To ensure accurate measurement, PPG assessment should be conducted under stable conditions, excluding sedation and ensuring hemodynamic stability. However, in clinical practice, PPG measurement often occurs directly at TIPS placement, which introduces potential biases such as the influence of sedatives like propofol and opioids, hemodynamic instability in patients with variceal bleeding, intubation, and the possible need for vasoactive medications (e.g., terlipressin, noradrenaline). Recognising these limitations, a recent study has proposed repeated PPG measurement after 24 h under stable conditions without sedation or intubation to yield more reliable results [3, 9]. Nevertheless, despite the potential benefits of delayed PPG measurement, there remains limited data on its clinical relevance and the subsequent adjustment of PPG.

Patients undergoing TIPS implantation are potential candidates for liver transplantation. However, due to organ shortage, it is imperative to identify individuals with poorer survival prospects following TIPS implantation, warranting early transplantation. Various parameters, such as The Model for End‐Stage Liver Disease (MELD) score and the FIPS score, were evaluated to identify high‐risk patients before TIPS implantation. The FIPS score, employed prior to TIPS implantation, can assist in categorising patients into TIPS and transplant groups [10, 11]. However, all these parameters rely on pre‐TIPS data, and dynamic changes during the procedure or post‐TIPS alterations have not been included so far. Nevertheless, owing to organ shortage, additional criteria are needed to identify patients within the TIPS group who may have worse survival outcomes.

Hence, the objective of our study was to ascertain factors associated with worse prognosis post‐TIPS implantation in a cohort of patients selected based on standard criteria, including the impact of PPG adjustment between the placement day and 24 h later on post‐TIPS survival.

## 2 Materials and Methods

We performed a prospective bicenter (University Hospitals Jena and Aachen) study including all consecutive patients who underwent TIPS procedure. Patients undergoing TIPS for the management of ascites or variceal haemorrhage (preemptive TIPS) were eligible for inclusion. Prior to TIPS, all patients underwent a comprehensive diagnostic evaluation including physical examination including vital parameters, laboratory testing, ECG, echocardiography, abdominal CT, abdominal ultrasound and measurement of HVPG. Data on demographics, medical history and routine clinical parameters and survival status were extracted from electronic patient records. The Model for End‐Stage Liver Disease (MELD) Score [12] and Child‐Pugh Score [13] were computed using standard laboratory parameters. All patients received regular visit in our outpatient clinic following TIPS insertion.

Ethical approval for the study was obtained from the local ethics committees (Jena University Hospital 2018‐1080‐BO, University Hospital RWTH Aachen EK023/19), and written informed consent was obtained from all patients before their inclusion in the study.

### 2.1 TIPS Procedure

Transjugular intrahepatic portosystemic shunt (TIPS; 8–10 mm Viator, W.L. Gore, Newark, Delaware, US) placement was conducted following local standard operating procedures, employing sedation (*n* = 84) with propofol, midazolam or pethidine and intubation (*n* = 21) as clinically indicated. Invasive measurement of portal and vena cava inferior pressures was performed using a pressure transducer system (Jena: Fa Braun, Melsungen, Germany; Aachen: CODAN pvb Critical Care GmbH, Forstinning, Germany). The portal pressure gradient (PPG) was determined as the difference between portal and vena cava inferior pressures. PPG measurements were obtained immediately after TIPS placement (PPG0). Additionally, 24 h post‐TIPS placement, invasive PPG measurement (PPG24h) was repeated without sedation and intubation, and the PPG difference (ΔPPG) was calculated from PPG24h and PPG0. Initial TIPS dilation on the day of implantation was limited to a maximum of 8 mm, with further dilation beyond this size performed only in select cases, such as those involving variceal bleeding. All patients underwent PPG measurement on the second day, and the pressure was adjusted to the target level if necessary. This approach was guide by the target PPG range 8–12 mmHg.

### 2.2 Blood Sampling

Blood samples were obtained from the cubital vein both before and 24 h following TIPS placement. Subsequently, they were centrifuged at 1800× *g* for 10 min. The resulting serum samples were then stored at −80°C until further analysis.

### 2.3 NT‐proBNP‐Measurement

NT‐proBNP was measured using an electrochemiluminescence immunoassay (Elecsys proBNP II) on a cobas 8000 e 801 immunoassay analyser (Roche, Mannheim, Germany) according to the manufacturer's recommendations.

### 2.4 Statistical Analysis

Statistical analysis was performed using SPSS v28 (IMB Inc., Armonk, NY, USA) and R 4.3.3. (www.r‐project.org) and graphic visualisations were created using PRISM 10 (GraphPad, LaJolla, CA, USA).

Statistical differences between unpaired data were assessed using the Mann–Whitney *U* test for continuous or ordinal data and Fisher's Exact test for nominal data. Statistical differences between paired data were analysed using the Wilcoxon Signed‐Rank test for continuous and ordinal data or the McNemar test for nominal data. Survival was assessed by Kaplan–Meier‐analysis and log‐rank test and was right‐censored at the event of liver transplantation, loss to follow‐up, or at 1 year, whichever occurred first. Competing risk analysis (Fine and Grey model) were performed, with death and liver transplantation treated as separate events. Univariate and multivariate logistic regression were conducted. Receiver operating characteristic (ROC) curves were generated and the area under the ROC curve (AUROC) was determined. Additionally, the Youden index was calculated to assess the diagnostic performance of the parameters. *p* < 0.05 in 2‐sided tests was considered statistically significant. Items with missing data were removed from the respective analysis.

## 3 Results

The baseline data of all patients (*n* = 105) are summarised in Table 1. Of the total cohort 67.7% (*n* = 71) had cirrhosis attributed to alcohol consumption. The median MELD score and median Child‐Pugh score for all patients were 11.0 (IQR 8.0; 15.0) and 8 (8; 9) points. The primary indication for TIPS implantation was refractory or recurrent ascites in 69.5% (*n* = 73) of cases, followed by variceal haemorrhage in 30.5% (*n* = 32). All included patients underwent TIPS implantation in accordance with international guidelines recommendations and met the recommended thresholds for parameters, such as total bilirubin levels [14] (Table 1). Patients were followed‐up for a median of 275 days. 11 patients (10.4%) died within 90 days, 22 patients died within 365 days (21.0%) and 6 patients (5.7%) underwent LTX within 1 year.

**TABLE 1:** Baseline data of the whole cohort and for patients with increased or lack of increased ΔPPG. Data are presented as median and 1st/3rd quartile or as absolute numbers and frequency.

Parameter	All *n* = 105	Lack of increased ΔPPG *n* = 39	Increased ΔPPG *n* = 66	*p* (lack of increased vs. increased)
Male (*n*=; %)	79 (75)	27 (71)	52 (77)	0.49
Age (mean ± SD; years)	59 (53; 63)	61.5 (54; 69)	57 (52; 65)	0.06
Ethology (*n*=; %) Alcohol/others	68 (64.8%)	21 (55.1%)	47 (70.1%)	0.44
Child‐Pugh points	8 (8; 9)	8 (7; 9)	8 (8; 9)	0.43
MELD	11 (8; 15)	11 (8; 16)	11 (7; 15)	0.84
FIPS	−0.25 (−0.79; 0.32)	−0.05 (−0.63; 0.54)	−0.32 (−0.92; 0.13)	0.19
Ascites (%)	73 (69.5%)	27 (71.1%)	46 (68.7%)	0.85
Variceal bleeding (%)	32 (30.5%)	11 (28.9%)	21 (31.3%)	0.79
Creatinine (μmol/L)	84.0 (65.3; 126.0)	87.5 (65.6; 152.1)	82.0 (64.0; 125.0)	0.32
Bilirubin (μmol/L)	20.0 (12.2; 34.5)	21.3 (12.5; 36.5)	19.0 (11.0; 33.0)	0.35
Albumin (g/L)	29 (26; 32)	31.0 (27.3; 36.5)	29.0 (24.0; 32.0)	0.01
ASAT (mmol/L)	0.69 (0.55; 0.89)	0.72 (0.53; 0.99)	0.68 (0.55;0.84)	0.56
ALAT (mmol/L)	0.33 (0.25; 0.51)	0.38 (0.27; 0.71)	0.33 (0.24; 0.46)	0.18
INR	1.3 (1.2; 1.5)	1.3 (1.1; 1.4)	1.4 (1.2; 1.5)	0.17
Platelet count	127 (85; 187)	156 (99; 193)	114 (79; 180)	0.09
C‐reactive protein (mg/L)	12.6 (7.1; 23.2)	15.7 (7.7; 34.0)	10.5 (5.5 21.3)	0.21
White blood cell count (Gpt/L)	8.4 (6.2; 11.2)	9.6 (7.3; 11.7)	8.0 (6.1; 11.2)	0.09
Baseline Nt‐proBNP (ng/L)	400 (151; 1074)	394 (155; 790)	420 (136; 1184)	0.72
PPG before TIPS (mmHg)	19 (15; 22)	20 (16; 27)	18 (15;22)	0.09
HF D0 (1/min)	75 (66; 83)	76 (62; 82)	75 (66; 84)	0.83
HF 24 h (1/min)	81 (69; 92)	83 (65; 90)	80 (69; 95)	0.73
RR D0 (mmHg)	110/66 (102/60; 122/76)	113/65 (102/59; 133/73)	110/66 (100/60; 119/78)	0.41
RR 24 h (mmHg)	105/58 (99/50; 116;63)	108/59 (95/50; 121/62)	104/57 (99/49; 114/66)	0.29
EF (%)	70.0 (65; 80)	73 (65; 80)	67.5 (64; 80)	0.34
Diastolic dysfunction (*n*=; %)	30 (28.6%)	7 (18.4%)	23 (34.3%)	0.52
Increased sPAP (*n*=; %)	11 (10.4%)	6 (15.8%)	5 (7.5%)	0.16

### 3.1 Changes in Portal Pressure Gradient 24 h After TIPS Implantation and Predictors of Survival

**FIGURE 1:**
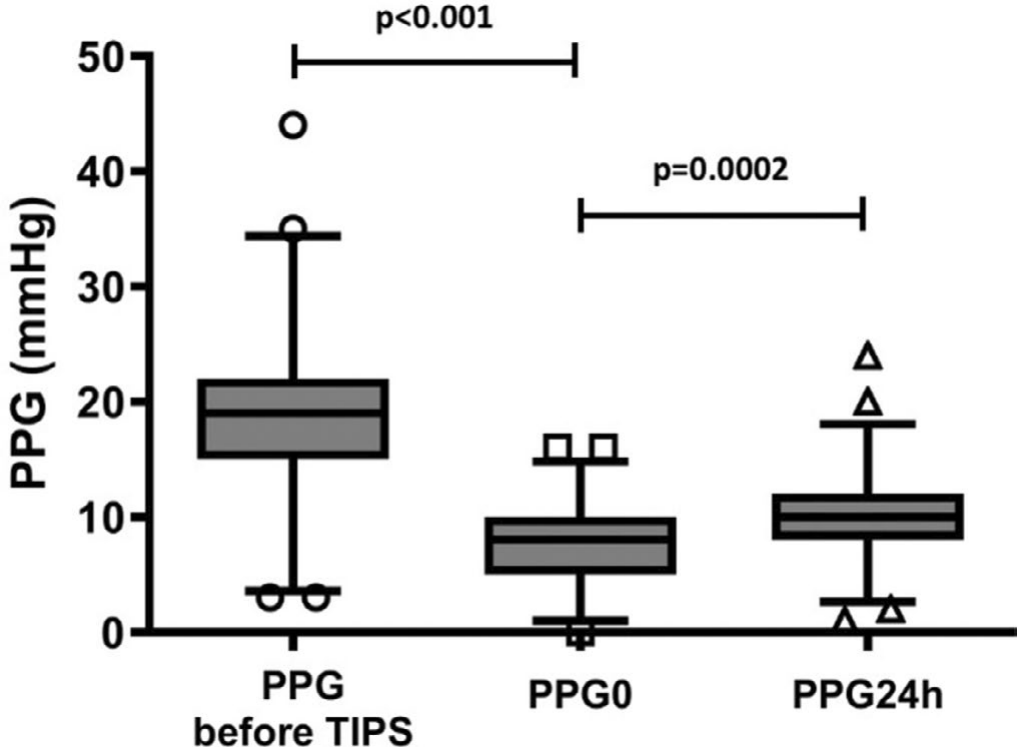
Absolute values of portal pressure gradient (PPG) in all patients before TIPS implantation, immediately after TIPS implantation (PPG0) and 24 h after TIPS implantation (PPG24h). Data are shows as median, IQR and 95% CI.

**FIGURE 2:**
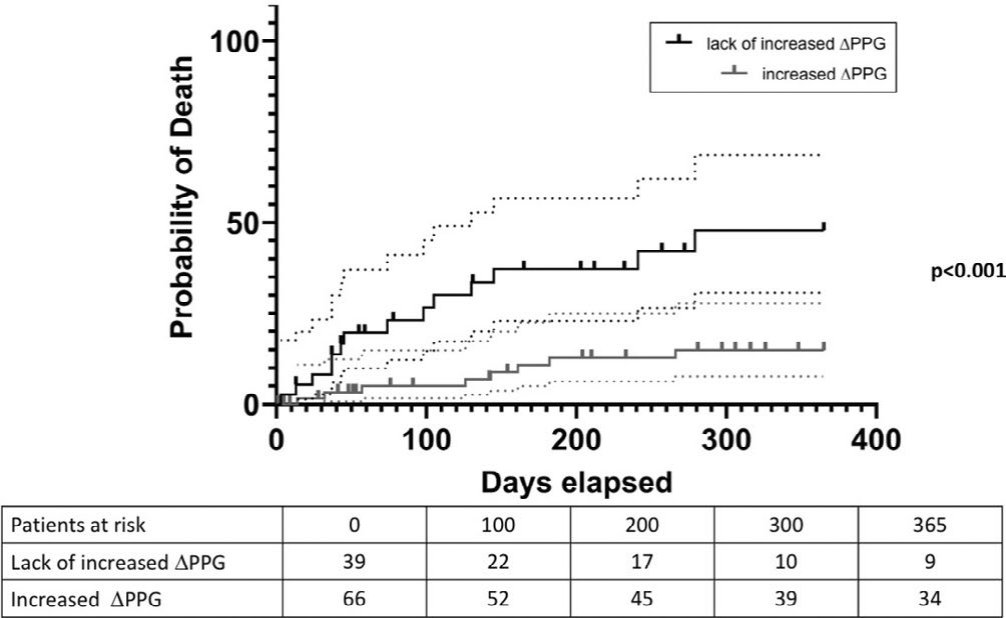
Probability of death of patients with lack of increased (black line) compared to increased ΔPPG (grey line) measured 24 h after TIPS implantation. Doted lines represent 95% CI.

Portal pressure gradient (PPG) was 19 (15; 22) mmHg before TIPS placement and 8 (5; 10) mmHg (PPG0; *p* < 0.0001) immediately after TIPS placement, indicating the anticipated decrease in portal pressure immediately post‐TIPS implantation on day 0. However, PPG measured 24 h later (PPG24h) was significantly higher than PPG0 (10 (8; 12) versus 8 (5; 10) mmHg; *p* = 0.0002; see Figure 1 and Figure S1). In total, a reintervention was indicated for *n* = 23 (21.9%) patients 24 h after TIPS implantation to reduce the PPG to target value. Notably, among the patients, *n* = 39 exhibited lack of increased ΔPPG values, which is unexpected given the absence of sedation. Interestingly, univariate logistic regression identified an association between 1‐year mortality and increased ΔPPG levels (OR 0.223, 95% CI 0.079–0.589, *p* = 0.003). Furthermore, we conducted a competing risk analysis, which revealed a subdistribution hazard ratio of 0.266 (95% CI 0.10–0.71; *p* = 0.008). Kaplan–Meier analysis showed poorer 90‐day and 1‐year cumulative estimates of survival among patients with lack of increased ΔPPG compared to those with increased ΔPPG values (90 days: 95 vs. 76.9%; *p* = 0.08; 1 year: 85.1 vs. 52.1%, *p* < 0.001) (Figure 2).

Additionally, besides lack of increased ΔPPG in univariate logistic regression, creatinine (OR 1.006, 95% CI 1.001–1.013, *p* = 0.041), FIPS > 0.92 (OR 6.094, 95% CI 1.463–27.108, *p* = 0.013) and total serum bilirubin levels (OR 1.013, 95% CI 1.003–1.029, *p* = 0.047) were associated with the 1‐year survival status. However, MELD score, Child‐Pugh score, and demographic factors did not exhibit a correlation with survival (all *p* > 0.05, data not shown). Furthermore, markers of cardiac dysfunction (such as NT‐proBNP and routine echocardiographic parameters) did not demonstrate an association with survival (data not shown). Given the limited occurrence of events, we conducted three distinct multivariable analyses, each incorporating variables ΔPPG and total bilirubin, creatinine or FIPS > 0.92, respectively (Table S1). Notably, in all three models, lack of increased ΔPPG demonstrated an independent association with survival. Furthermore, FIPS > 0.92 also emerged as independently associated with survival.

### 3.2 Laboratory Values and Hemodynamic Measurements Between Both Groups of ΔPPG

**FIGURE 3:**
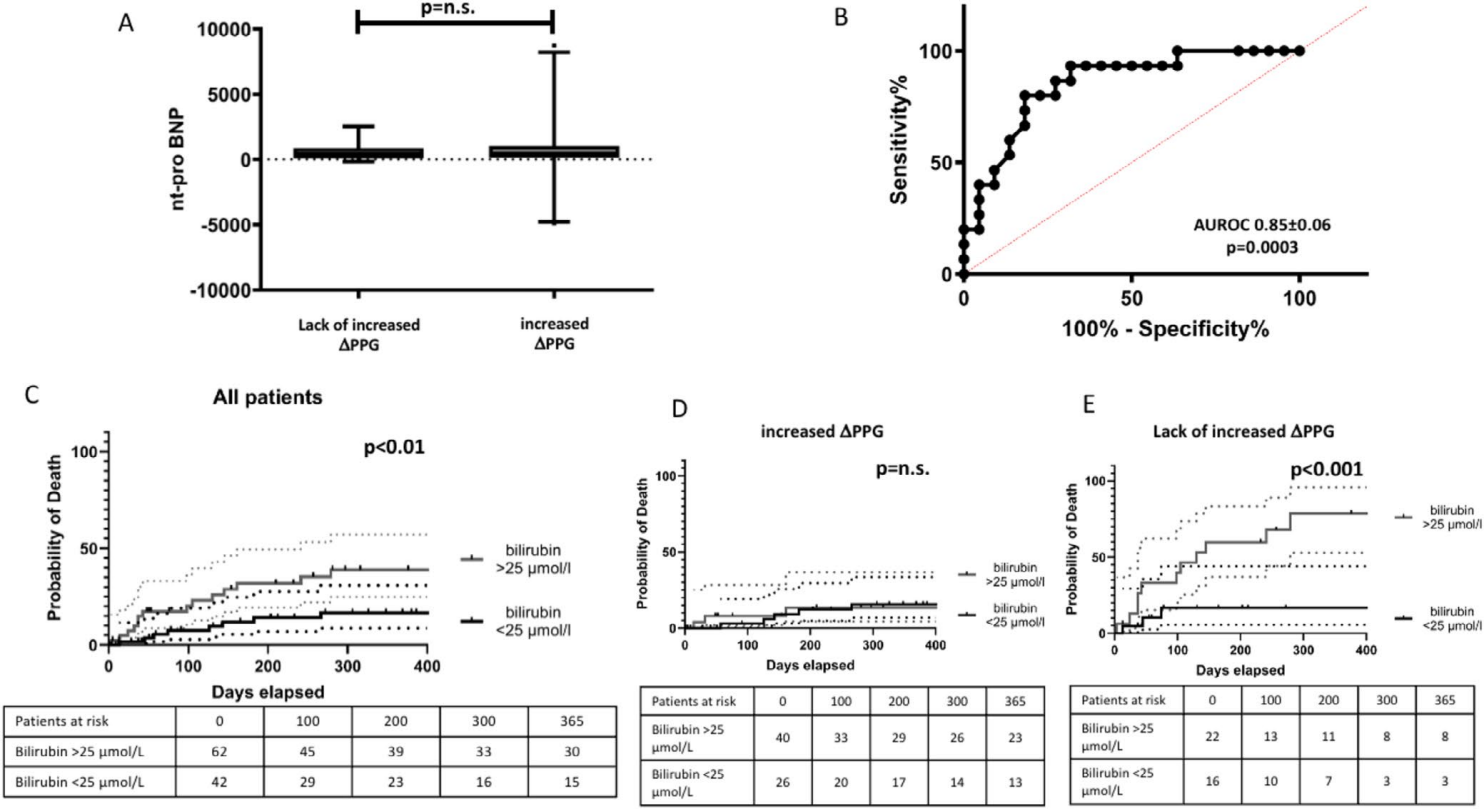
(A) Absolute values of NT‐proBNP in the groups of patients with lack of increased ΔPPG compared to increased ΔPPG. Data are shows as median, IQR and 95% CI. (B) Diagnostic accuracy of total serum bilirubin to predict death in the subgroups of patients with lack of increased ΔPPG (*n* = 39). (C–E) Kaplan–Meier survival curves with total bilirubin higher or lower than 25 μmol/L. (C) All patients. (D) Patients with increased ΔPPG. (E) Patients with lack of increased ΔPPG. Doted lines represent 95% CI.

Patients with lack of increased ΔPPG exhibited lower basal albumin levels (see Table 1). There were no statistically significant differences between the two groups regarding MELD, FIPS score, Child‐Pugh score, total bilirubin, serum creatinine, platelet count and INR measured before TIPS placement. Baseline NT‐proBNP levels and echocardiographic parameters, such as ejection fraction or diastolic dysfunction, did not show significant differences between the two groups. Subsequently, we compared NT‐proBNP levels 24 h and transaminases 24 h after TIPS implantation to identify short‐term differences between the groups. However, both parameters did not show significant differences between the groups. Additionally, we observed no significant difference between baseline (before TIPS implantation) and 24 h post‐TIPS implantation in NT‐proBNP and transaminase levels. The levels of NT‐proBNP are illustrated in Figure 3A.

Furthermore, there was no significant difference in PPG at 24 h or ΔPPG between patients who developed ascites after TIPS implantation (earliest occurrence after 4 weeks) and those who did not (Figure S2).

### 3.3 Prognostic Parameters to Predict Survival in the Group of Patients With Lack of Increased ΔPPG

Within the subgroup exhibiting lack of increased ΔPPG levels, a significant difference in total bilirubin levels before TIPS was observed between survivors and non‐survivors (16.0 vs. 36.0 μmol/L, *p* < 0.001), whereas no significant difference in bilirubin before TIPS was noted between survivors and non‐survivors among patients with increased ΔPPG (20 vs. 18.5 μmol/L, *p* = 0.537). The ROC curve analysis performed on patients with lack of increased ΔPPG in relation to bilirubin exhibited an area under the curve of 0.85 ± 0.06 (*p* = 0.0003, see Figure 3B), with a cut‐off value of over 25. Employing a bilirubin threshold of 25 μmol/L, we constructed 1‐year survival curves for all patients and their respective subgroups based on ΔPPG. These curves, depicted in Figure 3C–E, reveal a survival difference across the whole group of patients and among those with lack of increased ΔPPG levels, but not within the increased ΔPPG subgroup. Additionally, we assessed other parameters demonstrating significant associations with survival in univariate analysis. Neither creatinine nor FIPS > 0.92 exhibited a significant disparity between the two groups.

## 4 Discussion

In this prospective recruiting bicentric study, we show, for the first time, that hemodynamic changes that take place after TIPS, specifically changes in PPG, are associated to survival. Remarkably, this survival discrepancy remained independent of other established parameters associated with survival, such as MELD score [12], Child‐Pugh stage, FIPS > 0.92 [10], routine echocardiographic measurements [15], and NT‐proBNP values [16]. Hence, the maintenance or reduction of PPG measured 24 h post‐TIPS placement identifies a patient subgroup with markedly diminished survival prospects.

Predicting survival following TIPS placement remains challenging despite numerous proposed parameters for patient selection [10]. Despite careful patient selection, a subset of patients have poor outcomes after TIPS placement. Conceptually, both TIPS and liver transplant procedures can be considered for the same patients, making treatment decisions sometimes complex [11]. Established parameters like the MELD score often fail to distinguish between these both options, necessitating additional parameters to identify high‐risk patients with worse outcome [17]. Within our cohort, the median MELD score was below 12 in both groups. Patients with a MELD score of 14 points or lower exhibited minimal mortality [18]. Consequently, current EASL guidelines advocate for liver transplantation listing in individuals with a MELD score of 15 points or higher [19]. In our cohort, while adhering to proposed TIPS implantation criteria yielded a 1‐year survival rate of 79.0%, 10.4% of patients died within 3 months after TIPS implantation remained inadequately classified by MELD and FIPS [10]. Early detection of such high‐risk patients could facilitate timely intervention, such as liver transplantation. Our study's findings offer potential for identification of such individuals at higher risk post‐TIPS placement, who may urgently require liver transplantation.

The optimal timing for PPG measurement post‐TIPS remains a subject of ongoing debate. A recent study highlighted rapid hemodynamic changes following TIPS insertion, with authors noting fluctuations in cardiac output post‐sedation [20]. Despite immediate alterations post‐TIPS, these parameters continued to evolve up to 24 h afterward. Others has also advocated for PPG measurement 24 h post‐TIPS, considering the influence of sedation on PPG [9]. In our cohort, propofol was the primary sedative used, known for its association with vasoplegia and consequential hemodynamic shifts, such as vasodilation [21] and reductions in resistance index [22] and cardiac output [23]. Given the consistent vasoreactive effect of propofol regardless of dosage [24], we did not adjust for its quantity, administering it as clinically warranted. Conversely, one may opt for sedation using midazolam and ketamine instead of propofol. Despite lacking studies on the effects of these agents specifically on portal pressure, observations from hepatic venous pressure gradient (HVPG) measurements, a surrogate for portal pressure, caution against midazolam doses exceeding 0.03 mg/kg BW due to associated portal pressure alterations [25]. With this dosage, a patient weighing 80 kg would be permitted a maximum dose of 2.4 mg of midazolam, we postulate that even with the administration of these drugs, it may be challenging to completely avoid a reduction in portal pressure during TIPS implantation.

The inclusion of a 24‐h control was incorporated into our clinical protocol based on findings by Silva‐Junior et al. [9], which demonstrated optimal outcomes when measuring PPG at least 24 h post‐TIPS implantation in hemodynamically stable patients. It is worth noting that Silva‐Junior et al. conducted a relatively delayed third measurement at a median of 42 days post‐TIPS [9]. They did not observe any advantages from late measurements, we chose therefore not to introduce a third timepoint for PPG assessment. Consequently, there remains a possibility that patients may benefit from earlier assessments, such as at 7‐ or 28‐days post‐procedure.

Initial TIPS dilation on the day of implantation was limited to a maximum of 8 mm, with further dilation beyond this size performed only in select cases, such as those involving variceal bleeding. All patients underwent PPG measurement on the second day, and the pressure was adjusted to the target level if necessary. In light of the ongoing discussion regarding optimal TIPS diameters, our findings suggest that initially selecting smaller diameters (e.g., 6 mm or passive dilation only) may be a feasible strategy to prevent overtreatment, with adjustments made after the 24‐h evaluation. Evidence from a prospective, non‐randomised cohort study involving 42 patients with under‐dilated TIPS and 53 patients dilated to 8 mm supports the feasibility and effectiveness of under‐dilation [26]. Consequently, this approach warrants further investigation in prospective, randomised trials.

The pathophysiological mechanisms associated with the observed difference in survival is still unclear. We did not detect any association of PPG change with MELD, FIPS > 0.92, total bilirubin, NT‐proBNP and echocardiographic parameters like EF or the presence of increased systolic PAP or diastolic dysfunction. Besides the obvious hemodynamic changes of propofol the magnitude of these changes cannot be foreseen with the measured parameters. Interestingly, we found inside of the group of patients with lack of increased ΔPPG that total bilirubin with a threshold of 25 μmol/L further distinguish between survivors and non‐survivors with an AUROC of 0.85. This threshold was only relevant in the group of patients with lack of increased ΔPPG and further detected patients with worse survival.

Our study has some limitations. The absence of a later third measurement, as mentioned above, could potentially refine the results and identify patients experiencing changes in cardiac function following TIPS insertion. However, to date, no evidence has demonstrated that measurements taken beyond 24 h post‐TIPS insertion offer additional insights [9]. Thus, the utility of a later measurement, such as 7 days post‐implantation, remains speculative. Secondly, our study did not measure echocardiographic parameters 24 h after TIPS implantation and therefore cannot evaluate short‐term changes in cardiac function. Indeed, there are reports regarding echocardiographic parameters pre‐TIPS and their association with survival [27]. One of the most thoroughly evaluated parameter in this context is NT‐proBNP [16], which has the advantage of a short half‐life and rapid changes. However, this parameter did not differ among our patients and showed no association with survival. It is important to note that the endpoint in this study was cardiac failure, not survival. Furthermore, pre‐TIPS right atrial pressure (RAP) has been associated with worse outcomes following TIPS implantation [28]. In our study, we did not perform invasive RAP measurements; however, echocardiographic assessments did not indicate elevated RAP. A second measurement taken 24 h after TIPS insertion might provide a potential explanation for the observed lack of increase in PPG.

Thirdly, our findings lack external validation. Despite conducting a bicentric study, the limited number of included patients precludes a validation group. Nonetheless, we are currently undertaking a prospective, randomised, multicenter study across Germany, evaluating the effect of TIPS in patients with AKI‐HRS (LIVER‐HERO) [29]. In this study, we also perform a second measurement 24 h after TIPS insertion which will give further data.

In summary, our data demonstrate, for the first time, the clinical benefit of measuring PPG 24 h after TIPS insertion in identifying patients with poorer survival outcomes. Such patients may require more short‐term care. Consequently, we advocate for the implementation of a second measurement in patients undergoing TIPS insertion to optimise pressures in those with elevated pressure above the target PPG and to identify patients requiring heightened medical attention.

## Author Contributions

**P. A. Reuken:** writing – original draft, formal analysis. **A. Franz:** data curation. **T. H. Wirtz:** data curation. **C. Ripoll:** writing – review and editing. **R. Aschenbach:** writing – review and editing, investigation. **U. Teichgräber:** writing – review and editing, investigation. **M. R. Pollmanns:** data curation. **M. Kiehntopf:** investigation, writing – review and editing. **S. Keil:** investigation. **C. Kuhl:** investigation. **P. C. Schulze:** investigation. **C. Trautwein:** writing – review and editing. **T. Bruns:** writing – review and editing. **A. Stallmach:** writing – review and editing. **A. Zipprich:** conceptualization, writing – original draft, formal analysis, supervision.

## Ethics Statement

The study was approved by the local ethics committee of Jena University Hospital and RWTH Aachen.

## Consent

Written informed consent was given from every patients before inclusion.

## Conflicts of Interest

All authors report no conflict of interest regarding the content of the manuscript. Outside the submitted work, P.A.R. received lecture and consulting fees from Pfizer, BMS, Gilead, Advanz and CSL Behring. A.Z. received lecture and consulting fee from Falk, CSL Behring, Gore, Grifols, and Advanz. C.R. received lecture and/or consulting fees from Falk, CSL Behring, Gore, Grifols and Ingelheim‐Boehringer. TB received consulting fees from Intercept/Advanz Pharma, Grifols, and Sobi as well as honoraria for lectures, presentations, or educational events from Falk Foundation, CSL Behring, Merck, Gilead, Intercept/Advanz Pharma, and Gore.

## Supporting information


**Figure S1:** Comparison of PPG0, PPG24h and ΔPPG in patients who received sedation or intubation during TIPS‐procedure. Data are presented as median, IQR and 95% CI. Mann–Whitney test was applied to test statistical significance with *p < 0.05; ***p < 0.001.

**Figure S2:** Comparison of PPG24 and ΔPPG in patients had recurrent ascites with necessity of paracentesis after TIPS insertion. Data are presented as median, IQR and 95% CI. Mann–Whitney test was applied to test statistical significance.

**Table S1:** Results of the different models of the multivariate analysis.

## Data Availability

The data that support the findings of this study are available on request from the corresponding author. The data are not publicly available due to privacy or ethical restrictions.
