# Supplementary figures and images for: Early Dynamics of Portal Pressure Gradient After TIPS Insertion Predict Mortality
Source: Aliment Pharmacol Ther. 2025 Jan 16;61(7):1175–82. doi: 10.1111/apt.18503 (PMC11908110; doi:10.1111/apt.18503)

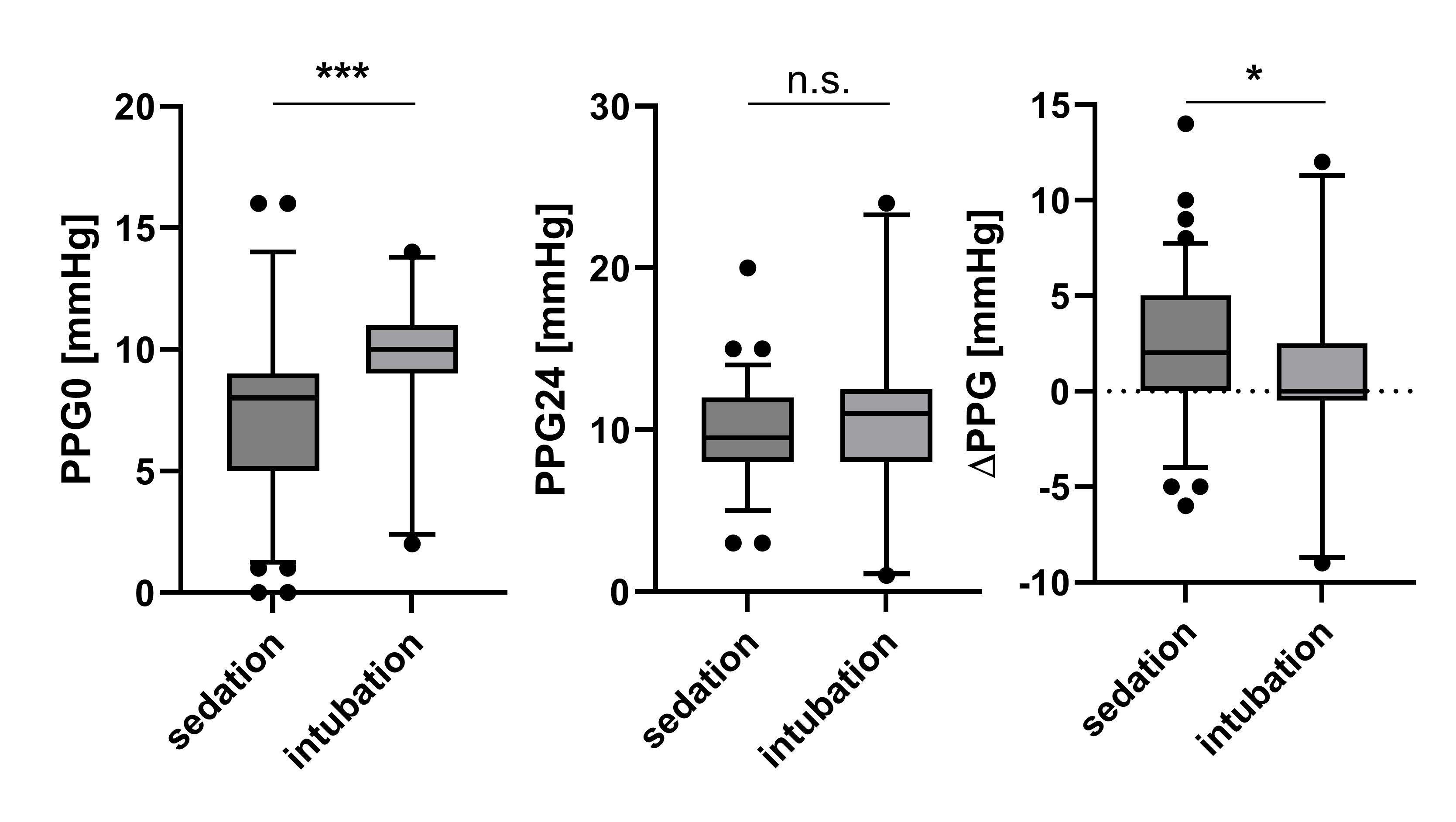

Supplement: Supplementary file 1 — Figure S1: Comparison of PPG0, PPG24h and ΔPPG in patients who received sedation or intubation during TIPS‐procedure. Data are presented as median, IQR and 95% CI. Mann–Whitney test was applied to test statistical significance with *p < 0.05; ***p < 0.001. [file APT-61-1175-s002.tif]

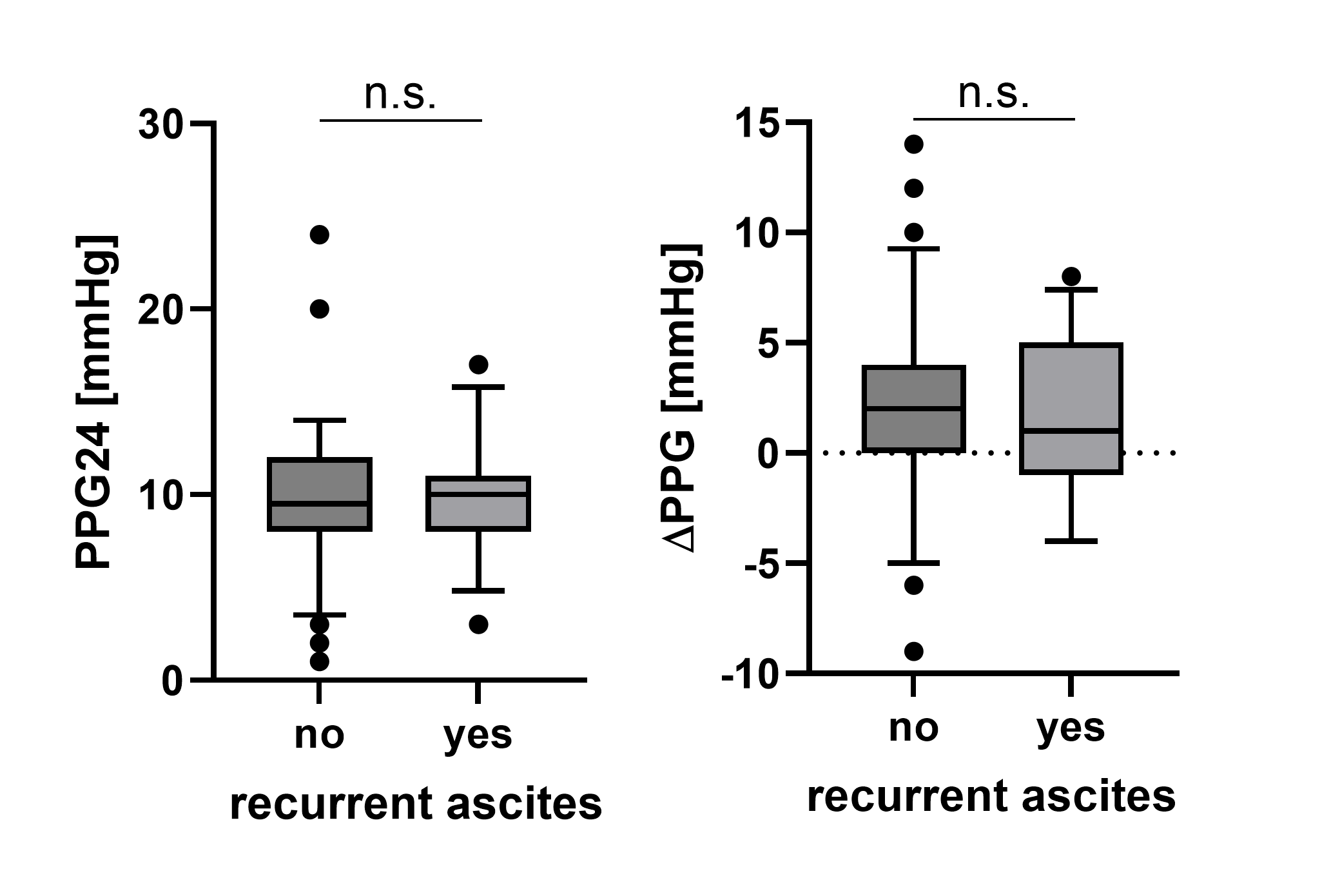

Supplement: Supplementary file 2 — Figure S2: Comparison of PPG24 and ΔPPG in patients had recurrent ascites with necessity of paracentesis after TIPS insertion. Data are presented as median, IQR and 95% CI. Mann–Whitney test was applied to test statistical significance. [file APT-61-1175-s003.tif]
