# Supplementary material for: Early Dynamics of Portal Pressure Gradient After TIPS Insertion Predict Mortality
Source: Aliment Pharmacol Ther. 2025 Jan 16;61(7):1175–82. doi: 10.1111/apt.18503 (PMC11908110; doi:10.1111/apt.18503)
Supplement: Supplementary file 3 — Table S1: Results of the different models of the multivariate analysis. [file APT-61-1175-s001.docx]

Suppl Table 1: Results of the different models of the multivariate analysis

| Included parameter | Odds ratio (95%CI) | P value |
| --- | --- | --- |
| FIPS | 5.25 (1.17 – 25.09) | 0.03 |
| ΔPPG >0 | 0.26 (0.09 – 0.72) | 0.01 |
|  |  |  |
| Bilirubin (µmol/L) | 1.01 (1.00 – 1.03) | 0.07 |
| ΔPPG >0 | 0.25 (0.09 – 0.69) | 0.009 |
|  |  |  |
| Creatinine (µmol/L) | 1.01 (1.00 – 1.01) | 0.07 |
| ΔPPG >0 | 0.24 (0.08 – 0.66) | 0.006 |
